# The Impact of Study Size on Meta-analyses: Examination of Underpowered Studies in Cochrane Reviews

**DOI:** 10.1371/journal.pone.0059202

**Published:** 2013-03-27

**Authors:** Rebecca M. Turner, Sheila M. Bird, Julian P. T. Higgins

**Affiliations:** 1 MRC Biostatistics Unit, Institute of Public Health, Cambridge, United Kingdom; 2 School of Social and Community Medicine, University of Bristol, Bristol, United Kingdom; 3 Centre for Reviews and Dissemination, University of York, York, United Kingdom; Copenhagen University Hospital Gentofte, Denmark

## Abstract

**Background:**

Most meta-analyses include data from one or more small studies that, individually, do not have power to detect an intervention effect. The relative influence of adequately powered and underpowered studies in published meta-analyses has not previously been explored. We examine the distribution of power available in studies within meta-analyses published in Cochrane reviews, and investigate the impact of underpowered studies on meta-analysis results.

**Methods and Findings:**

For 14,886 meta-analyses of binary outcomes from 1,991 Cochrane reviews, we calculated power per study within each meta-analysis. We defined adequate power as ≥50% power to detect a 30% relative risk reduction. In a subset of 1,107 meta-analyses including 5 or more studies with at least two adequately powered and at least one underpowered, results were compared with and without underpowered studies. In 10,492 (70%) of 14,886 meta-analyses, all included studies were underpowered; only 2,588 (17%) included at least two adequately powered studies. 34% of the meta-analyses themselves were adequately powered. The median of summary relative risks was 0.75 across all meta-analyses (inter-quartile range 0.55 to 0.89). In the subset examined, odds ratios in underpowered studies were 15% lower (95% CI 11% to 18%, P<0.0001) than in adequately powered studies, in meta-analyses of controlled pharmacological trials; and 12% lower (95% CI 7% to 17%, P<0.0001) in meta-analyses of controlled non-pharmacological trials. The standard error of the intervention effect increased by a median of 11% (inter-quartile range −1% to 35%) when underpowered studies were omitted; and between-study heterogeneity tended to decrease.

**Conclusions:**

When at least two adequately powered studies are available in meta-analyses reported by Cochrane reviews, underpowered studies often contribute little information, and could be left out if a rapid review of the evidence is required. However, underpowered studies made up the entirety of the evidence in most Cochrane reviews.

## Introduction

Systematic reviews of intervention studies aim to synthesise all available evidence meeting pre-specified eligibility criteria. Such criteria seldom address sample size. Meta-analyses may therefore include data from one or more small studies which, individually, do not have power to detect a modest intervention effect. Small studies tend to report greater intervention effects than larger studies [1]. So-called “small-study effects” may arise from reporting biases, whereby findings in smaller studies are more likely to be selected for publication on the basis of statistical significance [2]. Alternatively, small-study effects may arise from biases caused by methodological flaws arising more frequently in small studies [3], or may be due to true differences in the underlying effects between smaller and larger studies.

Some researchers argue for excluding small studies from meta-analyses. Specifically to reduce the effects of publication bias, Stanley suggested discarding 90% of the study estimates, so that conclusions are based on only the most precise 10% of studies [4]. Earlier, Kraemer proposed including only adequately powered studies in meta-analysis, both to remove publication bias and to discourage future researchers from carrying out small studies [5]. In teaching, Bird has long advocated that trials should not be started unless they could deliver at least 50% power in respect of a priori plausible, worthwhile effect sizes [6]. The prospect of inclusion in later meta-analyses may partly explain why investigators continue to feel justified in conducting underpowered studies [7]–[9]. Researchers who choose to undertake a study that is capable of detecting only an unrealistically large effect may lack understanding of both scientific methods and ethics [10].

Arguments for including small studies in meta-analyses uphold that evidence synthesis is best informed by all reasonably unbiased evidence and that no such evidence should be discarded lightly. Cut-offs based on study size, although scientifically cost-efficient, introduce an extra element of subjectivity and might not ameliorate bias if the remaining large studies are insufficiently critiqued [11]. Moreover, observing heterogeneity in effects across multiple independent trials is important, even if some of these are smaller, since this is likely to reflect heterogeneity that would occur in clinical practice [12]; [13]. Difficulties caused by reporting biases and related small-study effects can be addressed through statistical methods of adjustment [14]; [15].

In this paper, we explore the levels of power available in studies included in published meta-analyses, and examine the relative influence of adequately powered and underpowered studies on these meta-analyses.

## Methods

### Data

To examine power per study within meta-analyses and to explore whether this varies across different settings, we use evidence from the *Cochrane Database of Systematic Reviews* (*CDSR*: Issue 1, 2008), which was provided by the Nordic Cochrane Centre. Each meta-analysis was categorized by type of outcome, types of intervention compared, and medical specialty to which the research question related, as described elsewhere [16]. In this paper, we include all meta-analyses of binary outcomes that reported data from two or more studies (14,886 meta-analyses).

### Calculation of Power per Study

In meta-analysis *j*, power was calculated with respect to a fixed baseline event rate, 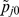. The median of the observed proportions experiencing events was calculated for each intervention arm separately and the higher median was used as 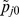. For each study *i* within meta-analysis *j* (with mean number of patients 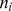 per treatment arm), we calculated how much power the study sample size provided to detect a relative risk reduction of 10%, 20%, 30% or 50% (or, equivalently, a relative risk of 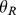 = 0.9, 0.8, 0.7 or 0.5). For convenience, we refer to a relative risk reduction of 30%, for example, as *RRR30*. In study *i* within meta-analysis *j*, the power to detect a difference between event rates 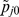 and 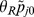 at a significance level of 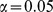 is given by:
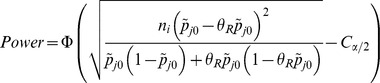
where 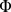 is the cumulative standard normal distribution function and 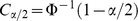. For our primary analyses we define adequate power as ≥50% power to detect *RRR30*. In subsequent analyses, we fitted a random-effects model to obtain a summary relative risk estimate, 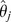, for meta-analysis *j*, and calculated the power of study *i* to detect the treatment effect observed in the meta-analysis to which it contributed, i.e. to detect a difference between 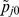 and 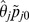.

### Calculation of power per Meta-analysis

The focus of this paper is on the power of primary studies within meta-analyses, but it is interesting also to examine the power of the meta-analyses themselves. In each meta-analysis *j*, we fitted a random-effects model, using a method-of-moments estimate for the between study variance [17], and calculated the variance *V_j_* of the combined intervention effect (on the log relative risk scale). The power of meta-analysis *j* to detect a 30% relative risk reduction or equivalently a log relative risk of 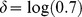, using a significance level of 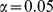 is given by:
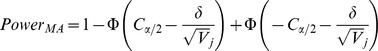
where 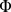 is the cumulative standard normal distribution function and 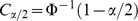 [18].

### Impact of Underpowered Studies

We defined subset A as *CDSR* meta-analyses that include five or more studies, with at least two adequately powered (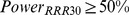) with respect to *RRR30* and at least one underpowered (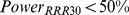), to investigate the impact of including or excluding underpowered studies. On the log odds ratio scale, per meta-analysis, we fitted fixed-effect and random-effects models including (1) all studies; (2) adequately powered studies only (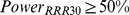) or (3) underpowered studies only (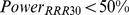).

For meta-analyses relating to beneficial rather than adverse outcomes, the data were rearranged, so that an odds ratio below 1 favours the experimental intervention over the comparator across all meta-analyses in subset A. A method-of-moments estimate was used for the between-study variance in the random-effects model [17].

As a descriptive analysis of the impact of excluding underpowered studies in subset A meta-analyses, we calculated ratios comparing meta-analysis results obtained from all studies with results from adequately powered studies only.

To compare effect sizes formally within subset A, we first estimated the average difference between log odds ratios in underpowered studies (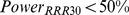) compared with adequately powered studies (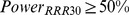) by fitting a random-effects meta-regression model. Then, in a random effects meta-analysis, we combined the estimated differences across subset A meta-analyses, with or without adjustment separately for (i) medical specialties, (ii) outcome type, (iii) intervention type. We also explored the role of underpowered studies in individual meta-analyses within a particular research setting in more detail, as described in Appendix S1.

## Results

### Power of Studies Included in Cochrane Reviews

**Figure 1:**
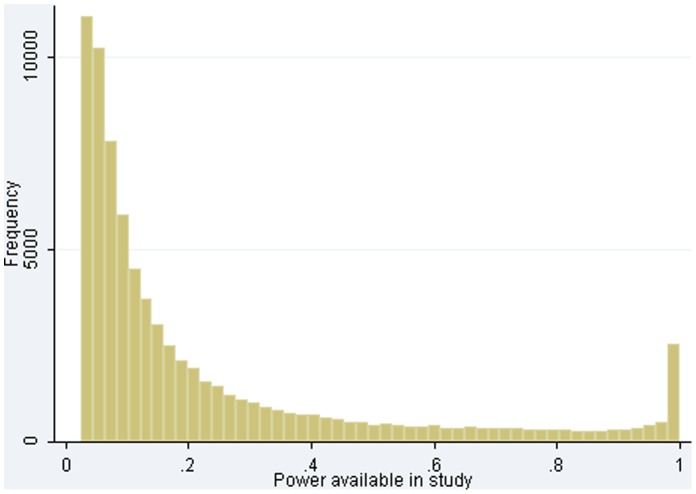
Distribution of power available to detect a relative risk reduction of 30%, across 77,237 studies.

Table 1 summarizes power of primary studies within meta-analyses in the *CDSR* database. In 10,492 (70%) of the 14,886 *CDSR* meta-analyses, all studies were underpowered (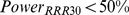) to detect a 30% relative risk reduction (*RRR30*). In many settings, a 20% relative risk reduction would be more realistic, and 85% of the meta-analyses included no studies powered to detect *RRR20*. Only 2,588 (17%) meta-analyses included at least two studies powered at 50% or more to detect *RRR30*, and only 1,291 (9%) included at least two studies powered at 80% or more. Median power within *CDSR* meta-analyses was low for *RRR30* at 13% power, with an inter-quartile range (*iqr*) of 7% to 31% power. Some studies were generously powered, with 2,571/77,237 (3.3%) having at least 98% power for *RRR30* (Figure 1).

**Table 1:** Percentages of 14,886 meta-analyses including no studies adequately powered to detect a target effect or including at least two adequately powered studies, where adequate power is defined as 80% or 50% in turn; and summary of median power within each meta-analysis.

Target effect	<80% powerin all studies	≥80% power inat least 2 studies	<50% power in all studies	≥50% power inat least 2 studies	Median (IQR) of median power within meta-analyses
10% relative risk reduction (*RRR10)*	98%	0.6%	96%	2%	0.05 (0.03 to 0.07)
20% relative risk reduction (*RRR20*)	92%	3%	85%	8%	0.08 (0.05 to 0.16)
30% relative risk reduction (*RRR30*)	83%	9%	70%	17%	0.13 (0.07 to 0.31)
50% relative risk reduction (*RRR50*)	62%	24%	46%	38%	0.31 (0.13 to 0.69)
Summary relative risk observed in meta-analysis	86%	8%	77%	15%	0.08 (0.04 to 0.26)

Power of studies to detect the summary relative risk in their meta-analysis was also low: 11,422 (77%) meta-analyses included no studies with ≥50% power and only 2,236 (15%) meta-analyses included at least two studies with ≥50% power. The median of summary relative risks was 0.75 across all meta-analyses (*iqr* 0.55 to 0.89).

Table 2 summarizes power for *RRR30* by medical specialty, outcome and intervention-comparison type. In cancer, 35% of 689 meta-analyses included at least two adequately powered studies, and only 365 meta-analyses (53%) consisted entirely of underpowered studies. However, median power within cancer meta-analyses remained low at 24% power (*iqr* 10% to 57% power).

**Table 2:** Numbers of adequately powered studies (≥50% power) and median power within each meta-analysis (MA) with respect to a 30% relative risk reduction (*RRR30*), overall and by medical specialty, outcome type and intervention-comparison type.

	N	% of MA in whichall studies underpowered	% of MA in which ≥2 studies adequately powered	Median (IQR) of median power within meta-analyses
All meta-analyses	14886	70%	17%	0.13 (0.07 to 0.31)
**Medical specialty**				
Cancer	689	53%	35%	0.24 (0.10 to 0.57)
Cardiovascular	1192	68%	19%	0.11 (0.06 to 0.26)
Central nervous system/musculoskeletal	1210	79%	11%	0.13 (0.06 to 0.26)
Digestive/endocr., nutritional and metabolic	1464	75%	16%	0.11 (0.05 to 0.28)
Gynaecology, pregnancy and birth	3905	72%	15%	0.11 (0.06 to 0.28)
Infectious diseases	780	62%	23%	0.16 (0.08 to 0.42)
Mental health and behavioural conditions	1977	73%	17%	0.14 (0.08 to 0.32)
Pathological conditions, symptoms and signs	414	64%	20%	0.17 (0.08 to 0.39)
Respiratory diseases	1310	75%	15%	0.12 (0.07 to 0.27)
Urogenital	932	77%	12%	0.12 (0.06 to 0.25)
Other medical specialties^1^	1013	61%	24%	0.18 (0.08 to 0.41)
**Outcome types**				
*Objective outcomes*				
All-cause mortality	1132	77%	14%	0.08 (0.05 to 0.18)
*Semi-objective outcomes*				
Obstetric outcomes	1288	71%	15%	0.12 (0.07 to 0.25)
Cause-specific mortality/major morbidity event/composite (mortality or morbidity)	907	76%	14%	0.08 (0.05 to 0.18)
Resource use/hospital stay/process	680	59%	22%	0.20 (0.08 to 0.42)
Other semi-objective outcomes^2^	1711	79%	12%	0.10 (0.06 to 0.22)
*Subjective outcomes*				
Adverse events	2330	81%	11%	0.11 (0.06 to 0.21)
Signs/symptoms reflecting continuation/end of condition	2184	54%	30%	0.25 (0.12 to 0.52)
Infection/onset of new acute/chronic disease	2038	75%	13%	0.11 (0.06 to 0.24)
Biological markers (dichotomised)	947	66%	21%	0.16 (0.07 to 0.39)
General physical health	276	75%	11%	0.13 (0.08 to 0.25)
Other subjective outcomes^3^	1331	59%	24%	0.22 (0.11 to 0.46)
**Intervention-comparison types**				
Pharmacological vs. Control/Placebo	5599	68%	18%	0.13 (0.07 to 0.31)
Non-pharmacological^4^ vs. Control/Placebo	2412	59%	26%	0.19 (0.08 to 0.47)
Active vs. Active	6875	76%	14%	0.11 (0.06 to 0.26)

1 Other medical specialties: Blood and immune system, Ear and nose, Eye, General health, Genetic disorders, Injuries, accidents and wounds, Mouth and dental, Skin.

2 Other semi-objective outcomes: External structure, Internal structure, Surgical/device related success/failure, Withdrawals/drop-outs.

3 Other subjective outcomes: Pain, Mental health outcomes, Quality of life/functioning, Consumption, Satisfaction with care, Composite (at least 1 non-mortality/morbidity).

4 Non-pharmacological interventions include interventions classified as medical devices, surgical, complex, resources and infrastructure, behavioural, psychological, physical, complementary, educational, radiotherapy, vaccines, cellular and gene, screening.

By outcome, we expected power to be lower for events that are typically rare. Power was indeed somewhat lower for meta-analyses reporting all-cause mortality and cause-specific mortality/major morbidity event/composite (mortality or morbidity), and somewhat higher for meta-analyses relating to resource use, signs/symptoms reflecting continuation/end of disease or a mixture of subjective outcomes (see Table 2).

### Power of Meta-analyses Included in Cochrane Reviews

Table 3 summarizes the power of the meta-analyses themselves to detect a 30% relative risk reduction, overall and by medical specialty, outcome and intervention-comparison type. Overall, the proportion of meta-analyses with 80% power or more to detect *RRR30* was 22%, with a further 12% powered at 50–80% to detect *RRR30*, but 66% were underpowered. At 34%, the proportion of adequately powered meta-analyses was substantially larger than the proportion of meta-analyses including at least two adequately powered studies, but remains low.

**Table 3:** Meta-analytic power with respect to a 30% relative risk reduction (RRR30), based on the random-effects model, overall and by medical specialty, outcome type and intervention-comparison type.

	N	% of MA in which meta-analytic power ≥50%	% of MA in which meta-analytic power ≥80%	Median (IQR) of meta-analytic power
All meta-analyses	14886	34%	22%	0.27 (0.11 to 0.72)
**Medical specialty**				
Cancer	689	51%	39%	0.51 (0.19 to 0.99)
Cardiovascular	1192	40%	28%	0.32 (0.12 to 0.86)
Central nervous system/musculoskeletal	1210	25%	13%	0.21 (0.10 to 0.50)
Digestive/endocr., nutritional and metabolic	1464	32%	21%	0.23 (0.10 to 0.68)
Gynaecology, pregnancy and birth	3905	31%	20%	0.23 (0.09 to 0.65)
Infectious diseases	780	35%	22%	0.27 (0.11 to 0.74)
Mental health and behavioural conditions	1977	38%	24%	0.32 (0.12 to 0.78)
Pathological conditions, symptoms and signs	414	37%	18%	0.31 (0.12 to 0.67)
Respiratory diseases	1310	34%	21%	0.28 (0.11 to 0.71)
Urogenital	932	27%	16%	0.24 (0.11 to 0.55)
Other medical specialties^1^	1013	39%	28%	0.35 (0.12 to 0.86)
**Outcome types**				
*Objective outcomes*				
All-cause mortality	1132	36%	25%	0.24 (0.10 to 0.81)
*Semi-objective outcomes*				
Obstetric outcomes	1288	38%	25%	0.31 (0.12 to 0.79)
Cause-specific mortality/major morbidity event/composite (mortality or morbidity)	907	33%	22%	0.22 (0.09 to 0.67)
Resource use/hospital stay/process	680	41%	27%	0.36 (0.12 to 0.83)
Other semi-objective outcomes^2^	1711	29%	18%	0.22 (0.10 to 0.59)
*Subjective outcomes*				
Adverse events	2330	24%	13%	0.19 (0.09 to 0.48)
Signs/symptoms reflecting continuation/end of condition	2184	46%	33%	0.42 (0.17 to 0.93)
Infection/onset of new acute/chronic disease	2038	28%	17%	0.22 (0.10 to 0.55)
Biological markers (dichotomised)	947	32%	21%	0.24 (0.10 to 0.69)
General physical health	276	29%	14%	0.26 (0.11 to 0.57)
Other subjective outcomes^3^	1331	45%	28%	0.41 (0.16 to 0.86)
**Intervention-comparison types**				
Pharmacological vs. Control/Placebo	5599	35%	22%	0.29 (0.12 to 0.73)
Non-pharmacological^4^ vs. Control/Placebo	2412	43%	28%	0.36 (0.13 to 0.87)
Active vs. Active	6875	30%	19%	0.23 (0.10 to 0.62)

1 Other medical specialties, semi-objective outcomes, subjective outcomes and non-pharmacological interventions defined in footnotes to Table 2.

The median of meta-analytic power was 27% (*iqr* 11% to 72% power). There was some variation across medical areas; in cancer, 51% of meta-analyses were powered at 50% or more. Differences in meta-analytic power across medical areas, outcome and intervention-comparison types were largely in the same direction as differences in meta-analysis summaries of study power (Table 2).

### Impact of Excluding Underpowered Studies from Meta-analyses

Of the 14,886 *CDSR* meta-analyses with binary outcomes, 1,107 (7.4%) were eligible for inclusion in subset A. The impact of excluding the underpowered trials on the results of these meta-analyses is summarised in Table 4. We calculated ratios comparing log odds ratio estimates from a meta-analysis of adequately powered studies only to those from a full meta-analysis. These are shown for fixed-effect and random-effects models separately.

**Table 4:** Ratios comparing results obtained from adequately powered studies only with results obtained from all studies, in subset A of 1,107 meta-analyses: results shown are percentiles of the distribution of such ratios across meta-analyses.

	Percentile				
	5%	25%	50%	75%	95%
Ratio of log OR estimates from fixed-effect (FE) meta-analysis, adequately powered studies only vs.all studies	−0.17	0.78	0.96	1.06	1.76
Ratio of log OR estimates from random-effects (RE) meta-analysis, adequately powered studies only vs. all studies	−0.40	0.67	0.94	1.10	1.85
Ratio of FE standard errors for log OR, adequately powered studies only vs. all studies	1.01	1.04	1.11	1.26	1.72
Ratio of RE standard errors for log OR, adequately powered studies only vs. all studies	0.63	0.99	1.11	1.35	2.20
Ratio of heterogeneity estimates, adequately powered studies only vs. all studies (where 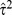 non-zero for all studies)^1^	0	0.04	0.79	1.18	2.81

1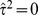 in the all-studies meta-analysis in 256/1107 meta-analyses. In 199/256 (78%), 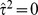 also in the meta-analysis including adequately powered studies only. In 57/256 (22%), 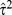 increased, but trivially, when underpowered studies were removed.

Across the 1,107 meta-analyses, there was a broad spread of ratios representing changes to the summary log odds ratio. The median ratio was 0.96 for the fixed-effect model and 0.94 for the random-effects model. The results correspond to a slight shift towards the null value when underpowered studies were removed, more so under the random-effects model in which small studies have greater influence.

Under the random-effects model, it is possible for precision to be gained (i.e. smaller standard error) when studies are removed, if the heterogeneity estimate is sufficiently reduced. The non-zero between-study heterogeneity in 851 meta-analyses decreased by a median of 21% when underpowered studies were removed (*iqr* −96% to +18%).

Table 5 presents average differences in log odds ratios between inadequately powered (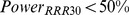) and adequately powered studies, obtained from fitting meta-epidemiological models to the subset of 1,107 meta-analyses. Overall, the difference was −0.10 (95% CI −0.13 to −0.08, P<0.0001), which corresponds to odds ratios in underpowered studies being 10% lower on average (95% CI 8% to 13%), where lower odds ratios represent more extreme effects in favour of the active treatment. There was evidence that differences in log odds ratios varied across medical areas (P = 0.001), and across intervention-comparison types (P = 0.0002), but not by outcome types (P = 0.83). By medical area, the greatest differences between inadequately and adequately powered studies were observed for infectious diseases, mental health and behavioural conditions, gynaecology, pregnancy and birth, and in the mixed subset of “other medical specialties” (defined in footnote to Table 2). In comparisons of two active interventions, the results are less meaningful since the direction of the intervention effect is likely to vary across meta-analyses in the data set. Odds ratios in underpowered studies were 15% lower (95% CI 11% to 18%, P<0.0001) in meta-analyses comparing pharmacological interventions against control or placebo, and 12% lower (95% CI 7% to 17%, P<0.0001) in meta-analyses comparing non-pharmacological interventions against control or placebo.

**Table 5:** Average differences in observed log odds ratios between underpowered (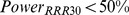) compared to adequately powered studies, in subset A of 1,107 meta-analyses, overall and within medical specialties, outcome types and intervention-comparison types.

	Difference in log OR (95% CI)	Between-meta-analysis standard deviation (95% CI)
**Overall**	−0.10 (−0.13, −0.08)	0.22 (0.22, 0.29)
**By medical specialty**		0.22 (0.21, 0.28)
Cancer	−0.01 (−0.08, 0.07)	
Cardiovascular	−0.12 (−0.18, −0.06)	
Central nervous system/musculoskeletal	0.04 (−0.08, 0.16)	
Digestive/endocr., nutritional and metabolic	−0.01 (−0.10, 0.08)	
Gynaecology, pregnancy and birth	−0.14 (−0.19, −0.09)	
Infectious diseases	−0.20 (−0.30, −0.09)	
Mental health and behavioural conditions	−0.15 (−0.22, −0.08)	
Pathological conditions, symptoms and signs	−0.03 (−0.18, 0.11)	
Respiratory diseases	−0.08 (−0.17, −0.002)	
Urogenital	−0.06 (−0.19, 0.06)	
Other medical specialties^1^	−0.21 (−0.30, −0.12)	
**By outcome type**		0.22 (0.22, 0.29)
All-cause mortality	−0.08 (−0.16, −0.003)	
Semi-objective outcomes^1^	−0.11 (−0.15, −0.06)	
Subjective outcomes^1^	−0.11 (−0.14, −0.08)	
**By intervention-comparison type**		0.21(0.21, 0.29)
Pharmacological vs. Control/Placebo	−0.15 (−0.18, −0.11)	
Non-pharmacological^1^ vs. Control/Placebo	−0.12 (−0.17, −0.07)	
Active vs. Active^2^	−0.03 (−0.07, 0.01)	

1 Other medical specialties, semi-objective outcomes, subjective outcomes and non-pharmacological interventions defined in footnotes to Table 2.

2 Comparison is less meaningful when comparing two active interventions since the a priori “better” active intervention is not taken into account.

In Appendix S1, the role of underpowered studies in individual meta-analyses is explored in more detail.

## Discussion

Underpowered studies made up the entirety of the evidence in most meta-analyses reported by Cochrane reviews: in 70% of *CDSR* meta-analyses, all studies had less than 50% power to detect a 30% relative risk reduction (*RRR30*), and only 17% of meta-analyses included at least two studies with at least 50% power for *RRR30*. There was some variation across medical areas and outcome types, but individual studies’ power was low across all types of meta-analyses.

In a meta-epidemiological analysis of 1,107 meta-analyses, we found that odds ratios in underpowered studies were on average 10% lower (95% CI 8% to 12%, P<0.0001) than those in adequately powered studies. This should be regarded as a lower limit on the difference, since the database contains treatment comparisons that have underlying relative risks either side of 1. Indeed, the difference was larger among comparisons involving a control or placebo group (15% for controlled pharmaceutical trials), in which we might expect the direction of effect to be more consistent across meta-analyses. In meta-analyses in which at least two adequately powered studies are available, underpowered studies often had relatively little impact on the summary estimate of the odds ratio. The summary estimate shifted slightly toward the null when underpowered studies were removed, under both fixed-effect and random-effects models. The extent to which precision was lost when underpowered studies were excluded varied across meta-analyses. Some meta-analyses included a few very large studies, which dominated their results, while in other meta-analyses all studies were similarly sized and exclusion of underpowered studies led to greater losses in precision.

On average, the between-study heterogeneity estimate decreased when underpowered studies were excluded from meta-analyses, which may be expected since underpowered studies tend to observe more extreme effect estimates. Within the subset of 1,107 meta-analyses examined, the heterogeneity estimate sometimes decreased substantially when underpowered studies were removed. However, we also found examples where the heterogeneity *increased* when underpowered studies were removed, in settings where, for example, the largest studies in the meta-analysis had produced extremely different results.

The meta-analyses themselves were better powered than the primary studies within meta-analyses, as we would expect: overall, 34% of *CDSR* meta-analyses had at least 50% power to detect *RRR30*. Elsewhere, in the setting of cumulative meta-analysis in particular, the information size required for a meta-analysis to detect a particular effect size has been used to examine whether meta-analyses contain enough information to be conclusive [19]–[21]. Our finding that 22% of 14,886 meta-analyses were powered at 80% or more for *RRR30* is comparable with, but much more precise than, the 39% of 174 meta-analyses from the Cochrane Neonatal Group, which were found to meet the information size criterion for *RRR30* with 80% power by Brok et al. [20], who had, however, excluded both reviews with fewer than three trials and those in which all trials had a high risk of bias, in which meta-analytic size is likely to have been smaller.

Our work is limited to meta-analyses from Cochrane reviews, which may not be representative of meta-analyses in general. In particular, the differences observed between medical areas may reflect differing advice or editorial policies between Cochrane Review Groups which oversee different medical areas rather than disease-specific differences.

Although underpowered when included in meta-analyses, some original studies may have been adequately powered for their own primary outcomes, since the results extracted for meta-analysis might have related to secondary outcomes. For example, a study designed to detect a difference in measures of depression would be unlikely to be adequately powered for all-cause mortality. We do not therefore intend to criticise authors of primary studies for the very low levels of power in these meta-analyses. Publication dates of primary studies were not always available in the *CDSR* database, and so we were unable to look at the association between study age and power. It is possible that studies carried out in more recent years were more generously powered. However, the reasons for lack of power in completed studies include over-enthusiasm of researchers for the effectiveness of a new intervention, problems with recruitment to the study, and inaccurate sample size calculations [22]; these issues are common in experimental research and unlikely to disappear.

It is well known that small studies included in a meta-analysis tend to show more extreme treatment effects than larger studies. The differences observed between underpowered and adequately powered studies in the *CDSR* data set are consistent with previous findings, but offer much greater precision. For example, in a combined analysis of 13 meta-analyses evaluating effects on pain in patients with osteoarthritis, Nüesch et al. [23] found an average difference of −0.21 (95% CI −0.34 to −0.08) in standardized mean differences, when comparing trials with fewer than 100 patients per arm with larger trials. Several methods have been proposed for addressing small study effects in meta-analysis; recently, these were reviewed by Sterne et al. [24], who published new guidelines.

The practical implications of our findings for systematic reviews and meta-analyses vary according to review purpose and the research time available. Systematic reviews commissioned to inform public health policy decisions, by the National Institute for Health and Clinical Excellence (NICE) for example, are often carried out to tight deadlines [25]. Where a rapid review of the evidence is required and if several large, high-quality studies have been found in initial searches, it may be justifiable to truncate the searching and perform the synthesis, since inclusion of more obscure, smaller studies is unlikely to change the conclusions of the review. On the other hand, many Cochrane reviews are carried out in areas of scientific uncertainty, where discrepancies exist between findings from previous, mainly small studies. Here, the objective of meta-analysis is to resolve uncertainty by combining all available evidence and investigating reasons for between-study heterogeneity, and it would be inappropriate to leave out smaller studies. When carrying out a rapid meta-analysis to inform a grant application, the appropriate choice is less clear; although smaller studies might add little information relative to the time required for data extraction, it may be unethical to randomise yet more patients if a meta-analysis including small, existing studies would provide conclusive evidence.

In conclusion, we found that underpowered studies play a very substantial role in meta-analyses reported by Cochrane reviews, since the majority of meta-analyses include no adequately powered studies. In meta-analyses including two or more adequately powered studies, the remaining underpowered studies often contributed little information to the combined results, and could be left out if a rapid review of the evidence is required.

## Supporting Information

Appendix S1 **Detailed exploration of the role of underpowered studies.**
(DOC)
