# Supplementary material for: The Impact of Study Size on Meta-analyses: Examination of Underpowered Studies in Cochrane Reviews
Source: PLoS One. 2013 Mar 27;8(3):e59202. doi: 10.1371/journal.pone.0059202 (PMC3609745; doi:10.1371/journal.pone.0059202)
Supplement: Appendix S1 — Detailed exploration of the role of underpowered studies. (DOC) [file pone.0059202.s001.doc]

**S1: Detailed exploration of the role of underpowered studies**

In additional analyses, we explored the role of underpowered studies in a subset of individual meta-analyses in more detail. We identified meta-analyses relating to cardiovascular disease, with outcomes of all-cause mortality, cause-specific mortality, major morbidity event or composite mortality/morbidity. As previously, we used only meta-analyses that include 5 or more studies, with at least two adequately powered ($Power_{RRR30}\geq50\%$) with respect to a 30% relative risk reduction (*RRR30*) and at least one underpowered ($Power_{RRR30}<50\%$). The *CDSR* database included 59 meta-analyses which met these criteria. We examined the results from fixed-effect and random-effects meta-analyses, comparing results obtained from all studies with results from adequately powered studies only.

In approximately half of the 59 meta-analyses, exclusion of inadequately powered studies ($Power_{RRR30}<50\%$) caused little change to the summary estimate of the odds ratio, while the 95% confidence interval widened. The extent of widening was influenced primarily by the weight given to adequately powered studies in the full meta-analysis. In some cases, adequately powered studies had contributed the majority of the evidence and therefore little precision was lost when underpowered studies were excluded. For example, in a Cochrane review examining the effectiveness of antiplatelet therapy in treating acute ischaemic stroke, nine randomised trials were included, with data on 41,399 patients in total, but two trials contributed 98% of the patients (Figure S1) [1]. For all-cause mortality, the odds ratio for antiplatelet therapy versus control was estimated as 0.92 (95% CI 0.85 to 1) when including all nine trials and as 0.92 (95% CI 0.83 to 1.02) when including only the two adequately powered trials. However, in meta-analyses where all studies were similarly sized, excluding studies below a cut-off of 50% power had more impact. For example, in a comparison of organised stroke unit care against general medical wards with respect to all-cause mortality in stroke patients, all 26 studies available had less than 60% power to detect *RRR30* [2]. Here, the odds ratio was estimated as 0.86 (95% CI 0.76 to 0.98) when including all 26 studies or as 0.80 (95% CI 0.64 to 1.00) when including only the three adequately powered studies (Figure S2).

In a few (3/59) meta-analyses, between-study heterogeneity reduced substantially when underpowered studies were excluded. As an example, a meta-analysis evaluating phosphodiesterase inhibitors (PDIs) for treatment of heart failure comprised nine studies [3]. Two of these studies included more than 1,000 patients, and the rest each included fewer than 500. The summary odds ratio estimate for cardiac death changed from 1.23 (95% CI 0.88 to 1.72) in the full meta-analysis to 1.25 (95% CI 1.03 to 1.52) in the meta-analysis including only the two adequately powered studies (Figure S3). Narrowing of the confidence interval was caused by reduction in heterogeneity from $\hat{\tau}^{2}=0.12$ (*I^2^*=64%) to $\hat{\tau}^{2}=0.007$ (*I^2^*=35%) when underpowered studies were removed. The Cochrane review authors discussed the high heterogeneity, which was primarily caused by the results of one underpowered trial (VSG 1993).

When high heterogeneity was present among effects reported by larger studies in the meta-analysis, exclusion of underpowered studies often led to changes in the summary estimate, as the relative weights given to the remaining studies were altered. In a meta-analysis evaluating the effect of antifibrinolytic drugs on cerebral ischaemia [4], heterogeneity was high in both the full meta-analysis including five randomised trials ($\hat{\tau}^{2}=0.17$, *I^2^*=57%) and the meta-analysis excluding three underpowered trials ($\hat{\tau}^{2}=0.18$, *I^2^*=79%). In this data set, the summary odds ratio estimate changed from 1.67 (95% CI 0.99 to 2.81) to 1.29 (95% CI 0.67 to 2.47) when underpowered trials were excluded (Figure S4). The high heterogeneity was discussed in the Cochrane review, and the authors noted that specific treatments to prevent cerebral ischaemia had been used in the most recent trial (Roos 2000).

References

[1] Sandercock P, Gubitz G, Foley P, Counsell C. Antiplatelet therapy for acute ischaemic stroke. Cochrane Database of Systematic Reviews 2008; **2**.

[2] Stroke Unit Trialists' Collaboration. Organised inpatient (stroke unit) care for stroke. Cochrane Database of Systematic Reviews 2009; **1**.

[3] Amsallem E, Kasparian C, Haddour G, Boissel JP, Nony P. Phosphodiesterase III inhibitors for heart failure. Cochrane Database of Systematic Reviews 2009; **1**.

[4] Roos YB, Rinkel GJE, Vermeulen M, Algra A, van Gijn J. Antifibrinolytic therapy for aneurysmal subarachnoid haemorrhage. Cochrane Database of Systematic Reviews 2008; **4**.

**
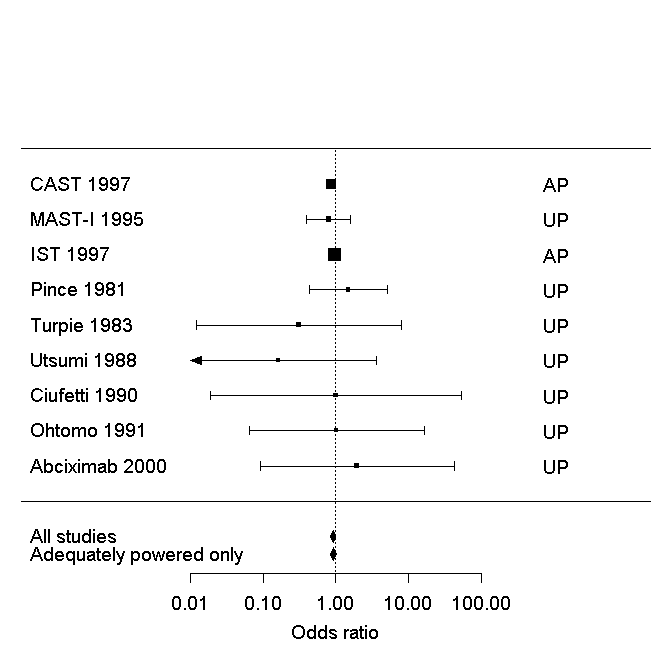
**

**Figure S1** Meta-analysis comparing the effectiveness of antiplatelet therapy in treating acute ischaemic stroke: (a) including all trials, both adequately powered (AP) and underpowered (UP); (b) including adequately powered trials only ($Power_{RRR30}\geq50\%$) [1].

**
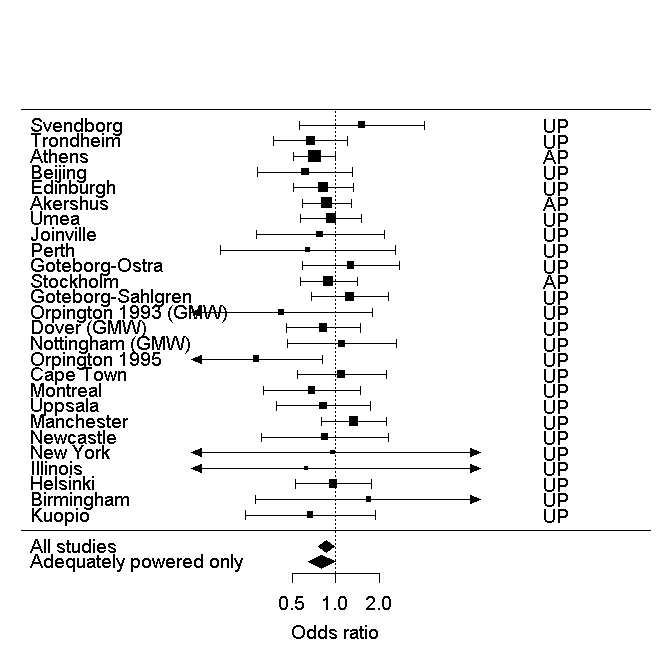
**

**Figure S2** Meta-analysis evaluating the effectiveness of organised stroke unit care vs. general medical wards for prevention of all-cause mortality in stroke patients: (a) including all trials, both adequately powered (AP) and underpowered (UP); (b) including adequately powered trials only ($Power_{RRR30}\geq50\%$) [2].


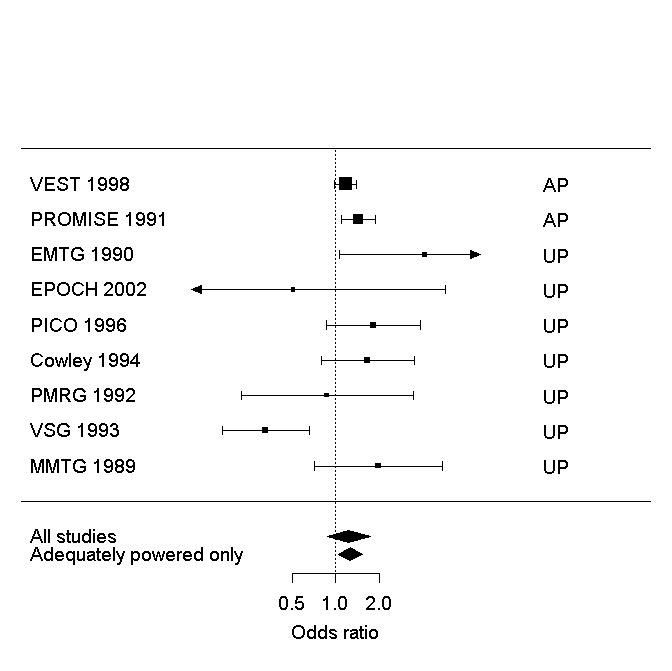


**Figure S3**  Meta-analysis comparing the effect of phosphodiesterase inhibitors (PDIs) vs. placebo on cardiac death: (a) including all trials, both adequately powered (AP) and underpowered (UP); (b) including adequately powered trials only ($Power_{RRR30}\geq50\%$) [3].


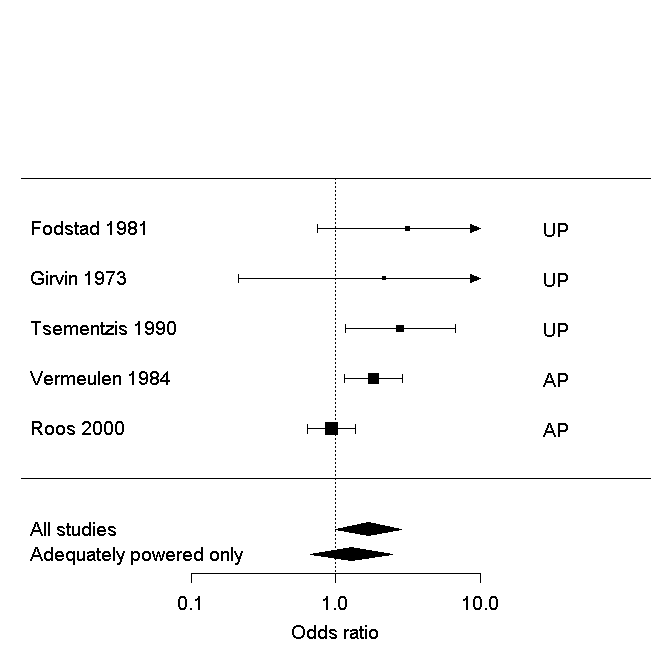


**Figure S4** Meta-analysis comparing the effect of antifibrinolytic drugs vs. control treatment on cerebral ischaemia: (a) including all trials, both adequately powered (AP) and underpowered (UP); (b) including adequately powered trials only ($Power_{RRR30}\geq50\%$) [4].
